# Modulating *miRNA-367-3p* Expression by Kaempferol Alleviates Experimental Autoimmune Encephalomyelitis: Targeting Fpn1-Dependent Ferroptosis and cAMP/CREB/CNTF Signaling

**DOI:** 10.1007/s11064-025-04641-2

**Published:** 2026-01-14

**Authors:** Rehab M. El-Gohary, Heba M. Shoeib, Ramez A. E. Barhoma, Shimaa M. Badr, Shaimaa Mohammed Zaher, Rehab E. Abo El Gheit, Ola A. Elshora, Mona H. Elamly, Mostafa Rizk Magar, Gamaleldien Elsayed Abdelkader, Asmaa S. Mohamed

**Affiliations:** 1 Medical Biochemistry Department, Faculty of Medicine, Tanta University, Tanta, 31527 Egypt; 2 Medical Physiology Department, Faculty of Medicine, Tanta University, Tanta, Egypt; 3 Department of Restorative Dentistry and Basic Medical Sciences, Faculty of Dentistry, University of Petra, Amman, 11196 Jordan; 4 Histology and cell biology department, Faculty of medicine, Tanta University, Tanta, Egypt; 5 Histology and cytology department, Faculty of medicine, Helwan University, Cairo, Egypt; 6 Clinical and Chemical Pathology Department, Faculty of Medicine, Tanta University, Tanta, Egypt; 7 Medical Pharmacology Department, Faculty of Medicine, Tanta University, Tanta, Egypt; 8 Department of Anatomy and Embryology, Damietta Faculty of Medicine, Al-Azhar University, Damietta, 34517 Egypt

**Keywords:** Multiple sclerosis, Kaempferol, miRNA-367-3P, Ciliary neurotrophic factor, Ferroportin1, Ferroptosis

## Abstract

Multiple sclerosis (MS) is a progressive, immune-mediated demyelinating disorder of the central nervous system (CNS). Kaempferol (KAM), a dietary bioflavonoid found in many edible and medicinal plants, exhibits significant neuroprotective effects in various immunological and neurological disorders; however, its therapeutic potential in MS remains largely unexplored. This study aimed to investigate the protective effects of KAM and the underlying molecular mechanisms using an experimental autoimmune encephalomyelitis (EAE) mouse model of MS. 40 female C57B1/6 mice were assigned to 4 groups: Normal control [saline (i.d.) + DMSO (i.p.)]; KAM [saline (i.d.) + KAM (50 mg/kg/d, i.p.)]; EAE [MOG^35–55^ immunization (i.d.) + DMSO (i.p.)]; and EAE + KAM [MOG^35–55^ immunization (i.d.) + KAM (50 mg/kg/d, i.p.)]. The brain and spinal cord were dissected for biochemical, molecular, histopathological, electron microscopic, and immunohistochemical analysis. KAM administration efficiently reduced clinical scores and ameliorated neural cytomorphological abnormalities. KAM profoundly combated iron overload and effectively upregulated ferroportin1 (Fpn1)-encoding gene expression. Furthermore, KAM valuably counteracted neuronal ferroptosis chiefly by restoring the Slc7A11/GSH/GPX4 axis. KAM considerably attenuated proinflammatory cytokine IL-17 and chemokine CCL-19. Intriguingly, KAM promoted axonal remyelination as indicated by an observable escalation in myelin basic protein content through activating the cAMP/CREB/ciliary neurotrophic factor (CNTF) axis. Collectively, for the first time, these findings demonstrated KAM’s neuroprotective potency against EAE, considering its antioxidant, anti-ferroptotic, immunomodulatory, anti-inflammatory, and neurotrophic properties, primarily mediated by inhibiting Fpn1-mediated ferroptosis, activating the cAMP/CREB/CNTF axis, and enhancing *miRNA-367-3p* expression. Accordingly, miRNA-367-3p has been proposed as an upcoming therapeutic target for MS, and KAM could be a promising treatment option for MS patients.

## Introduction

Multiple sclerosis (MS) is one of the most prevalent chronic non-traumatic causes of neurologic disability in adults worldwide. It is a neurodegenerative condition characterized by progressive loss of myelin sheaths surrounding neurons of the central nervous system (CNS) and axonal damage driven by autoreactive immune cells [1–4]. The incidence of MS is consistently higher in women than in men, with global female-to-male ratios ranging between 2:1 and 3:1. The disease exhibits distinct sexual dimorphism: although women are more frequently affected, men tend to experience later onset and more severe disease progression. This disparity arises from multifactorial causes, including the influence of sex hormones and genes located on the female sex chromosomes [5].

The interaction between genetic factors and epigenetic modifications plays a significant role in the pathophysiology of MS [6]. Despite advances in therapy, current treatment approaches remain unable to halt the progressive destruction of nervous tissue. Therefore, identifying innovative and safe compounds that specifically target the underlying mechanisms of the disease is of paramount importance for developing more effective interventions.

Recently, evidence has indicated the presence of iron deposition in the CNS of MS patients, suggesting an association between iron accumulation, neuroinflammatory changes, and oxidative tissue damage, which ultimately contributes to demyelination and neurodegeneration [7]. Ferroptosis is a distinct iron-dependent, non-apoptotic form of regulated cell death characterized by the accumulation of lipid peroxides [8]. The latest evidence suggests that ferroptosis may be involved in neuronal cell death associated with the development of multiple neurological diseases, including MS. This process is marked by excessive lipid peroxidation, mitochondrial dysfunction, and decreased activity of glutathione peroxidase 4 (GPX4) [9].

Ferroportin 1 (Fpn1), encoded by solute carrier family 40 member 1 (SLC40A1), is the sole mammalian iron exporter identified to date and functions by transporting iron from the cytoplasm to the extracellular environment [10]. Recent investigations have shown that Fpn1 is downregulated in neurodegenerative diseases [11, 12]. Nevertheless, the precise role of Fpn1 in brain iron dysregulation and functional impairment of MS remains elusive.

Cyclic adenosine monophosphate (cAMP) has been identified as one of the most extensively studied second messengers, playing a potent negative regulatory role in immune cell function. In vitro studies have shown that elevated cAMP levels enhance axon regeneration [13]. Noticeably, ciliary neurotrophic factor (CNTF), a pluripotent neurotrophic factor secreted by astrocytes, promotes neurogenesis and axonal regeneration and plays a crucial role in protecting oligodendrocytes from various forms of cell death. Moreover, CNTF facilitates the maturation of oligodendroglial precursor cells into myelin-producing cells, thereby promoting efficient myelination [14]. Consequently, CNTF upregulation represents a promising therapeutic strategy for managing MS.

Growing evidence suggests that dysregulation of microRNAs (miRNAs) plays a significant role in the pathogenesis of MS, highlighting brain miRNAs as potential therapeutic targets for MS treatment [6]. miR-367-3p is a class of endogenous non-coding miRNA (21 nucleotides) that is widely expressed in eukaryotic cells [15]. Importantly, miR-367-3p serves as a critical regulator of post-transcriptional gene networks in the brain, selectively targeting mRNAs and influencing the expression of associated proteins [16]. Downregulation of miR-367-3p has been linked to increased neuroinflammation, neuronal damage, and microglial activation [16, 17]. Interestingly, Fan et al. [18] reported that exogenous administration of miR-367-3p may alleviate MS pathology by attenuating microglial ferroptosis. Therefore, targeting miR-367-3p represents a promising therapeutic strategy for managing various CNS disorders, including MS.

Natural product-based foods and compounds have recently attracted considerable interest. They have promising neuroprotective properties, high tolerable doses, and are safe to use. Among such compounds, kaempferol (KAM; 3,4′,5,7-tetrahydroxyflavone), which is a naturally occurring bioactive flavonoid widely found in numerous edible and medicinal plants, such as fruits, vegetables, and traditional herbs, has garnered particular attention [19]. KAM has diverse beneficial effects, based on its anti-inflammatory, anti-aging, antioxidant, cytoprotective, and immunomodulatory properties [20]. Moreover, KAM has been reported to be an effective therapeutic modality in the management of autoimmune neural disorders [21]. Recently, Liu et al. [22] reported that KAM can mitigate motor disability and attenuate myelin loss in cuprizone-induced demyelination by suppressing STAT3 phosphorylation and NF-κB signaling.

Although KAM exhibits notable advantages in neuroprotection against multiple neurodegenerative disorders, including Parkinson’s and Alzheimer’s diseases [19, 23], its neuroprotective potential against experimental autoimmune encephalomyelitis (EAE), a typical murine model of MS, remains uncertain. Hence, the current study is the first to investigate whether KAM influences EAE by attenuating Fpn1-mediated ferroptosis and activating the cAMP/CREB/CNTF axis. To underline its related molecular mechanisms involved, we explored whether KAM’s effects on neuroinflammation and demyelination in EAE are mediated through modulating *miR-367-3p* expression.

## Materials and Methods

### Chemicals

Recombinant mouse myelin oligodendrocyte glycoprotein (MOG)^35–55^ was obtained from Cambridge Research Biochemicals (Billingham, UK). Incomplete Freund’s adjuvant (IFA, F5506) and kaempferol (KAM, CAS no.: 520-18-3; purity ≥ 90%) were purchased from Sigma-Aldrich (St. Louis, Missouri, USA). KAM powder was dissolved in 10% (*w/v*) dimethyl sulfoxide (DMSO) as the vehicle. All other chemicals, unless otherwise stated, were acquired from Sigma-Aldrich and were of a high analytical grade.

### Experimental Animals

Forty female C57BL/6 mice (**RRID: IMSR_JAX:000664**), aged 8–10 weeks, were obtained from the Research Center of Experiments, Tanta University. Animals were housed in wire mesh cages under standard laboratory conditions with a 12-hour light/dark cycle, controlled temperature (22 ± 2 °C), and humidity (50 ± 10%). Mice had ad libitum access to standard chow and water throughout the experimental period. All experimental procedures were approved by the Medical Research Ethics Committee, Faculty of Medicine, Tanta University (Approval Code: 36264PR1221/5/25) and were conducted in compliance with the ARRIVE guidelines 2.0 for animal research [24].

### Establishment of the EAE Model

Following one week of acclimatization, EAE was induced in designated mice according to the protocol described by El-Deeb et al. [25]. For induction, mice received a single intradermal (i.d.) injection at the base of the tail containing 200 µL of inoculum prepared by emulsifying 20 µg of recombinant mouse MOG^35–55^ in saline (1:1, *v/v*) with an equal volume of IFA. This immunization procedure was designated as Day 0 of the experiment. Control mice received a single i.d. injection of 0.2 mL saline instead.

### Experimental Grouping and Treatment Schedules

One week after the adaptive feeding, the 40 mice were randomly assigned to four experimental groups (*n* = 10 per group):

Group I: Normal control (NC): Healthy mice received a single i.d. injection of 0.2 mL saline at the base of the tail on day 0. Beginning on day 10, mice received daily intraperitoneal (i.p.) injections of vehicle (10% DMSO) until the end of the experimental period.
Group II: Kaempferol control (KAM) group: Healthy mice received a single i.d. injection of 0.2 mL saline on day 0. Starting from day 10, mice received daily i.p. injections of KAM (50 mg/kg) until the end of the experiment (6 weeks).
Group III: EAE disease model (EAE) group: Mice were immunized with MOG^35–55^/IFA emulsion on day 0 to induce EAE. From day 10 post-immunization, mice received daily i.p. injections of vehicle (10% DMSO) until the end of the experiment.
Group IV: (EAE + KAM)-treated group: Mice were immunized with MOG^35–55^/IFA emulsion on day 0. From day 10 post-immunization, mice received daily i.p. injections of KAM (50 mg/kg) until the end of the experiment (6 weeks).

### The Rationale for KAM Dose Selection

The KAM dose of 50 mg/kg was selected based on a comprehensive review of previous preclinical studies demonstrating optimal neuroprotection and immunomodulation at this dosage. Specifically, this dose has been shown to effectively improve the blood-brain barrier integrity, exert antioxidant and anti-inflammatory effects [26, 27], and improve neurological outcomes in various experimental models without observable toxicity [19, 21]. The daily i.p. administration route was chosen to maintain consistent systemic bioavailability, ensuring adequate systemic and CNS exposure.

### Clinical Assessment and Follow-Up

Mice were monitored daily in a double-blind manner throughout the experimental period for signs of disease development and progression. Motor deficits and neurological symptoms were evaluated weekly using Kono’s standardized 5-point scoring system, with intermediate scores of 0.5 used when symptoms fell between defined criteria, as detailed in Table 1 [28]. This scoring system evaluates disease severity based on the degree of motor impairment and paralysis manifestations.

**Table 1:** Clinical scoring criteria [28]

Score	Scoring standard	
0	No symptoms	
1	Tail drooping and weakness.	
2	Unilateral hindlimb paralysis	
3	Bilateral hindlimbs paralyzed, unable to turn over.	
4	Quadriplegia or urinary incontinence	
5	Near death or death.	

### Tissue Sampling

After six-week post-immunization, mice were anesthetized via intraperitoneal injection of pentobarbital (40 mg/kg) and subsequently sacrificed by decapitation. Whole brain and spinal cord tissues were dissected and rinsed in phosphate-buffered saline (PBS, pH 7.4).

The brain (cerebral cortex) and spinal cord were then divided into four parts. The first part was preserved in 10% formol saline solution for 24 h and then processed for histopathological examination and immunohistochemical study. The second part was preserved in glutaraldehyde for transmission electron microscope study. The third part was homogenized in 50 mM phosphate buffer saline, pH 7.4. The tissue homogenates were then centrifuged at 4000 rpm for 15 min. The supernatant was collected and frozen at − 80 °C, avoiding repeated freeze-thaw cycles, for further biochemical study, and total protein content was assessed according to the Bradford technique [29]. Meanwhile, the remaining part was frozen at − 80 °C for molecular study.

### Biochemical Assays

#### Assessment of Ferroptosis and Redox Status Markers

Brain and spinal cord Fe^2+^ were assessed using a commercial ferrous iron assay kit (Cat. No: E-BC-K304-S) purchased from Elabscience Biotechnology Co., USA. In this method, Fe^2+^ is released via the addition of an acidic buffer, followed by its reaction with a bipyridine chromogen to yield a measurable color, and its absorbance was measured at 450 nm.

Reduced glutathione (GSH) level was measured spectrophotometrically in brain and spinal cord tissue samples using a Bio-diagnostic commercial kit (Cat. No: GR2511; Giza, Egypt) according to Ellman’s protocol [30]. The principle of the assay is based on the reduction of DTNB (5,5’-dithiobis-(2-nitrobenzoic acid)) by glutathione (GSH), yielding a yellow-colored product, and its absorbance was measured at 405 nm.

Brain and spinal cord malondialdehyde (MDA) levels were quantified spectrophotometrically using the MDA-specific Bio-diagnostic kit (Cat. No: MD2529; Giza, Egypt). In this assay, MDA reacts with thiobarbituric acid (TBA) in an acidic medium to generate a pink chromophore, the absorbance of which is measured at 534 nm and is proportional to the MDA level in the sample as described by Ohkawa et al. [31].

Brain and spinal cord SLC7A11 and GPX4 levels were assessed by mouse ELISA kits purchased from FineTest, Wuhan, China (Cat. No: EM8310) and MyBioSource (San Diego, CA, USA, Cat. No: MBS909843).

#### Assessment of Interleukin (IL)-17 and C-C Motif Chemokine Ligand-19 (CCL-19)

Brain and spinal cord IL-17 and CCL-19 levels were assessed by mouse ELISA kits purchased from MyBioSource, San Diego, CA, USA (Cat. Nos: MBS2508197 and MBS177585, respectively), according to supplied manuals.

#### Assessment of cAMP and Phosphorylated (p)-cAMP Response Element Binding Protein (p-CREB) Levels

cAMP and p-CREB were estimated in brain and spinal cord tissue homogenates by mouse ELISA kits purchased from MyBioSource (San Diego, CA, USA, Cat. No: MBS265450 and MBS7254617, respectively), according to supplied manuals.

### Quantitative PCR for *SLC40A1*, *CNTF*, and *miR-367–3p* Expression

Stored neuronal tissue samples were used for isolation of total RNA (including microRNA) by using the microRNA Neasy Mini Kit (Catalog Number #217004) obtained from QIAGEN in the USA. RNA concentration and purity were verified using a NanoDrop spectrophotometer (NanoDrop Technologies, Inc., Wilmington, NC, USA) by measuring the OD260/280 ratio. For mRNA, cDNA was synthesized from total RNA using Revert Aid H Minus Reverse Transcriptase (Thermo Scientific, #EP0451, Waltham, MA, USA) and then used as a template for SYBR-green qPCR using a Step One Plus real-time PCR system (Applied Biosystem, USA). Values of *SLC40A1* (Fpn1-encoded gene) and *CNTF* were normalized to the GAPDH housekeeping gene. For miRNA, the TransScript^®^ Green microRNA Two-Step qRT-PCR Super Mix kit (Catalog Number #AQ202-01) from Transgen Biotech was utilized for reverse transcription of microRNA, and then quantitative RT-PCR was conducted for the expression of *miR-367–3p*. The expression was quantified relative to the small nuclear RNA *U6* housekeeping gene. Relative gene expression levels were calculated by normalizing the cycle threshold (Ct) values of target genes to the Ct value of an endogenous housekeeping gene control according to the fold change Livak method [32]. Table 2 displayed the sequences of the primer used.

**Table 2:** The primer sequences used in qRT-PCR

Gene	Primer sequence	GenBank Accession number
*Fpn1* *(Slc40A1)*	F: 5′-TGGATGGGTCCTTACTGTCTGCTAC‐3ʹ R: 5ʹ-TGCTAATCTGCTCCTGTTTTCTCC‐3ʹ	NM_016917.2
*CNTF*	F: 5ʹ-TGCTCTAGAATGGCTTTCGCAGAGCAA‐3ʹ R: 5ʹ-CTTGCGGCCGCCTACATTTGCTTGGCCCC‐3ʹ	NM_170786.2
*GAPDH*	F: 5′-GTGTGAACGGATTTGGCCGTATTGGGCG-3′ R: 5′-TCGCTCCTGGAAGATGGTGATGGGC-3′	NM_001289726.2
*miR-367–3p*	F: 5′-TGGAATTGCACTTTAGCAATG-3′ R:5′-CTCAACTGGTGTCGTGGAGTC-3′	NR_030268.1329
*RNA U6*	F: 5ʹ-CTCGCTTCGGCAGCACAT‐3ʹ R: 5ʹ-AACGCTTCACGAATTTGCGT‐3ʹ	NM_197993.4

### Histopathological Examination

Cerebral cortex and spinal cord samples were fixed, dehydrated, and cleared. After embedding, sections of 5 microns were stained with hematoxylin and eosin (H&E).

### Immunohistochemistry Examination

5 μm thick sections of the cerebral cortex and spinal cord were deparaffinized, rehydrated, rinsed, and incubated with the primary antibody (myelin basic protein (MBP) to demonstrate the myelin membrane) (Bioss Antibodies, USA, catalog number bs-0380r, dilution 1:200; **RRID: AB_2835364**). Thereafter, they were co-incubated with biotinylated secondary antibody (goat anti rabbit IgG, PE conjugated (bs-0295G-PE; **RRID: AB_10894102)** at 1:200 for 40 min at 37°). Immunohistochemical activity was visualized by 3, 3′-diaminobenzidine chromogen. Mayer’s hematoxylin was added as a counterstain in some sections. Saline was used as a substitute for the primary antibody to provide the negative control. The positive control for MBP was brain tissue. A positive reaction appeared as brown membranous.

The Image J software (National Institute of Health, Bethesda, Maryland, USA; **RRID: SCR_003070)** was used for image analysis. Three non-overlapping high-power fields from each slide of 5 rats/group were examined to count the mean number of inflammatory cells /mm^2^ for both cerebral cortex and spinal cord specimens. Ten separate non-overlapping fields from each group were examined to quantify the mean area percentage of MBP in immunohistochemically stained sections at a magnification of x400 for cerebral cortex and spinal cord specimens. Ten separate non-overlapping fields from each group were examined to quantify the mean area percentage of MBP in immunohistochemically stained sections at a magnification of X400 for cerebral cortex and spinal cord specimens.

### Transmission Electron Microscope (TEM)

Cerebral cortex and spinal cord specimens were finely cut and immersed in 4% phosphate-buffered glutaraldehyde (0.1 mol/L, pH 7.4) as a primary fixative, then post-fixed using 1% osmium tetroxide. Subsequently, specimens were dehydrated by using ascending grades of alcohol. Then, sections were embedded in a mixture of epoxy resin. Semi-thin and ultrathin sections were cut. Ultrathin sections were double stained using lead citrate and uranyl acetate to be studied and photographed by transmission electron microscope (JEOL-JEM-100, Tokyo, Japan) at the Electron Microscopy Unit, Faculty of Medicine, Tanta, Egypt.

### Statistical Analysis

Data normality was checked before data analysis using the Shapiro-Wilk test. For non-parametric data (clinical scores), the Mann Whitney *U* test was used to compare two groups. For parametric data, a one-way analysis of variance (ANOVA) was used to compare more than two groups, followed by a Tukey post-hoc test for multiple intergroup comparisons. A *P-*value of < 0.05 was deemed significant.

## Results

### KAM Attenuated EAE Clinical Neurological Signs

**Fig. 1:**
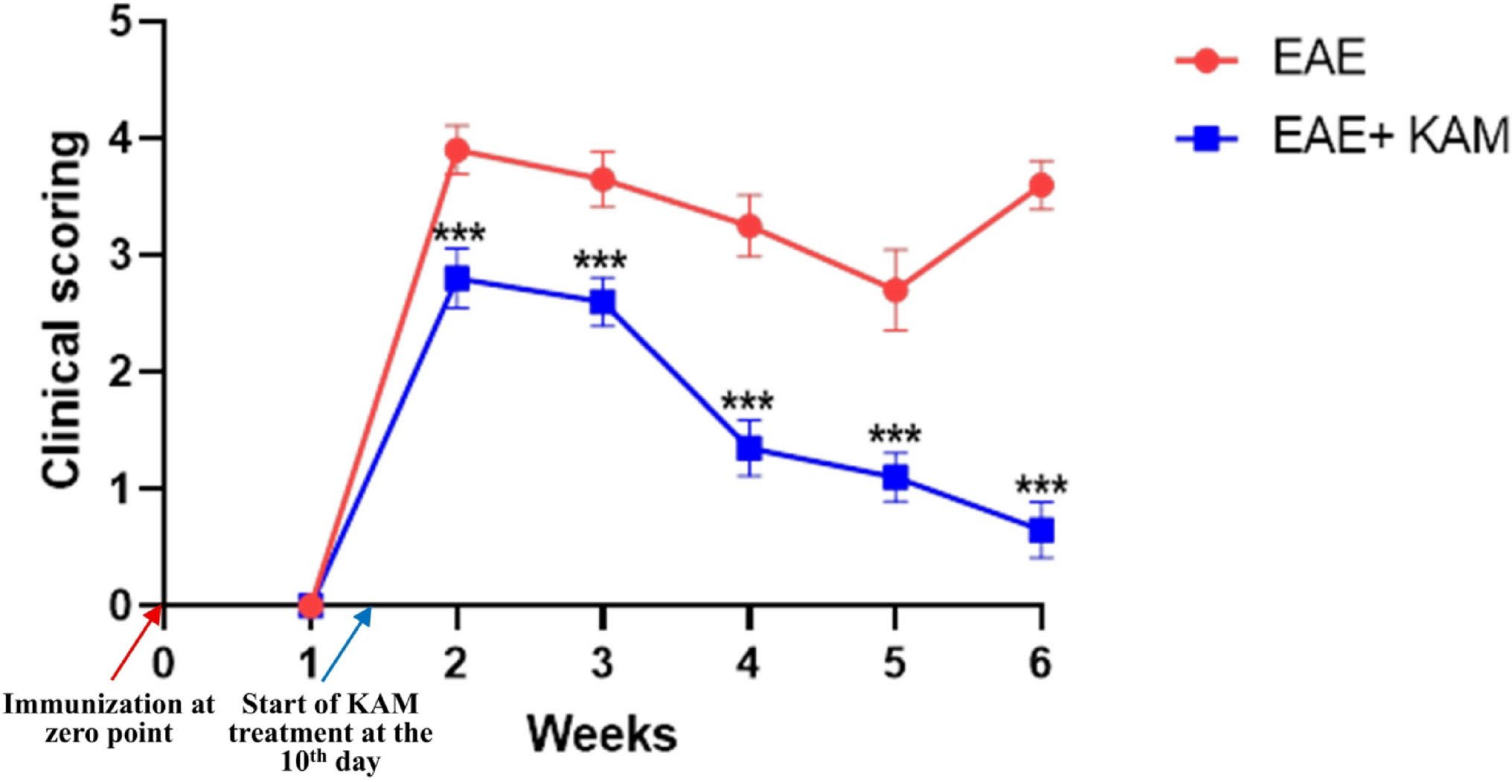
Progress of the clinical disability score in experimental autoimmune encephalomyelitis (EAE group; red line) vs. EAE treated by kaempferol (EAE + KAM group; blue line) to detect difference at each weekly recording points. Values were presented as mean ± SD (*n* = 10). ***p < 0.001, by Mann-Whitney U test

To investigate the neuroprotective effect of KAM on MS pathogenesis, (8–10)-week-old female C57BL/6 mice were immunized with MOG^35–55^ peptide to establish the EAE model (a valid model of MS) and then treated with KAM. Clinical scoring was used to assess neurological dysfunction across different groups. As shown in Table 3; Fig. 1, the untreated immunized EAE group exhibited a peak clinical score at the beginning of the second week, reaching 3.9 ± 0.21. This was followed by a gradual decrease (remission) to a mean of 2.7 ± 0.35 by the 5th week, with a subsequent relapse by the 6th week to a mean score of 3.6 ± 0.31. In contrast, the EAE + KAM group showed that KAM treatment (50 mg/kg) significantly reduced the peak clinical disability score (maximum score: 2.8 ± 0.26), accelerated remission, and prevented relapse in the 6th week, with a considerably lower score of 0.65 ± 0.24. These results indicated that KAM effectively alleviated the severity of clinical symptoms in EAE mice and halted disease progression.

**Table 3:** Weekly-based clinical scores recorded post-immunization in EAE and EAE + KAM groups

Week	Mean ± SD		Z of Mann Whitney U test	*P* value
	EAE	EAE + KAM		
1	0	0	0.000	1.0000
2	3.9 ± 0.21	2.8 ± 0.26	-3.979	< 0.0001*
3	3.65 ± 0.24	2.6 ± 0.21	-4.004	< 0.0001*
4	3.25 ± 0.26	1.35 ± 0.24	-3.930	< 0.0001*
5	2.7 ± 0.35	1.1 ± 0.21	-3.999	< 0.0001*
6	3.6 ± 0.31	0.65 ± 0.24	-4.004	< 0.0001*

*Statistically significant SD standard deviation

### KAM Ameliorated Histopathological Alterations in the Brain and Spinal Cord of EAE Mice

**Fig. 2:**
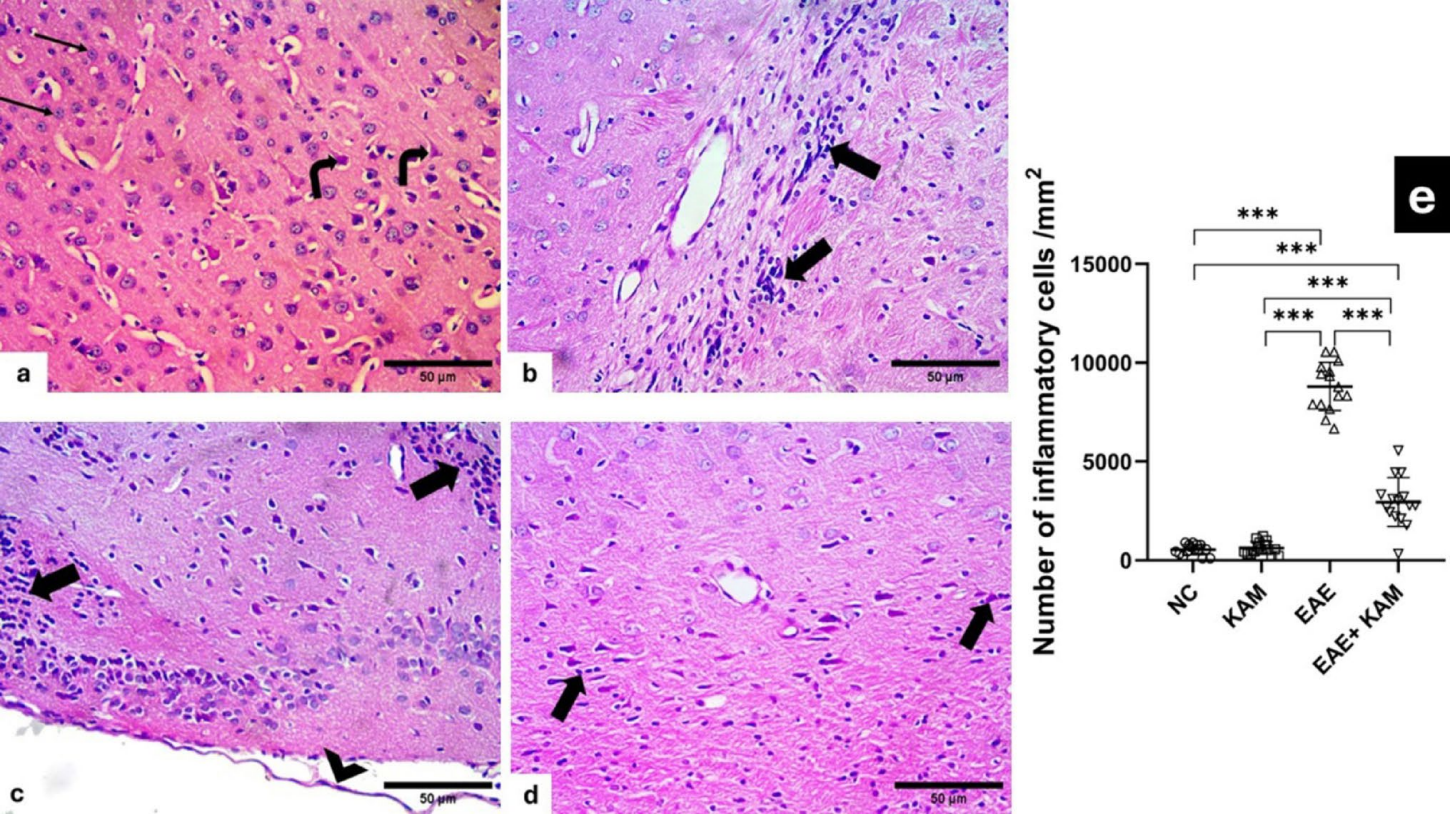
Effect of KAM (50 mg/kg) treatment on brain (cerebral cortex) histopathological alteration in the EAE model of MS. **a** Control of the cerebral cortex showing normal pyramidal cells (angled arrows) with their apical dendrite, granule cells with pale nuclei and prominent nucleoli (arrows) separated by eosinophilic neuropil. (**b** & **c**) EAE group with marked inflammatory cellular infiltration forming perivascular cuffs within the layers (thick arrows). Also, separated overlying meninges are present (arrowhead). **d** The KAM-treated group shows improvement with few inflammatory infiltrates (thick arrows). **e** Comparison between the studied groups as regards the mean number of inflammatory cells/mm² in cerebral cortex sections. (Magnification: X400, H&E, scale bar: 50 µ). NC: normal control; KAM: kaempferol; EAE: experimental autoimmune encephalomyelitis

H&E-stained sections from the control groups of the cerebral cortex showed the normal structure of cerebral cortex neurons, like pyramidal cells with their pyramidal shape and apical dendrite, granule cells with its pale nuclei and prominent nucleoli, separated by compact eosinophilic neuropil containing blood capillaries (Fig. 2a). The EAE group examination depicted severe focal disruption and marked inflammatory cellular infiltrations within the molecular layer, other layers, and surrounding the dilated blood capillaries forming perivascular cuff with separated meninges (Fig. 2b&c) and depicted an extremely significant increase in the mean number of inflammatory infiltrate/mm^2^ as compared to the control group (F (3,56) = 283, *P* < 0.0001, Fig. 2e). It is noteworthy that administration of KAM alleviated most of the previous changes except for the presence of some inflammatory infiltrates and a significant decrease in the number of inflammatory infiltrates as compared to the EAE group (Fig. 2d&e).

**Fig. 3:**
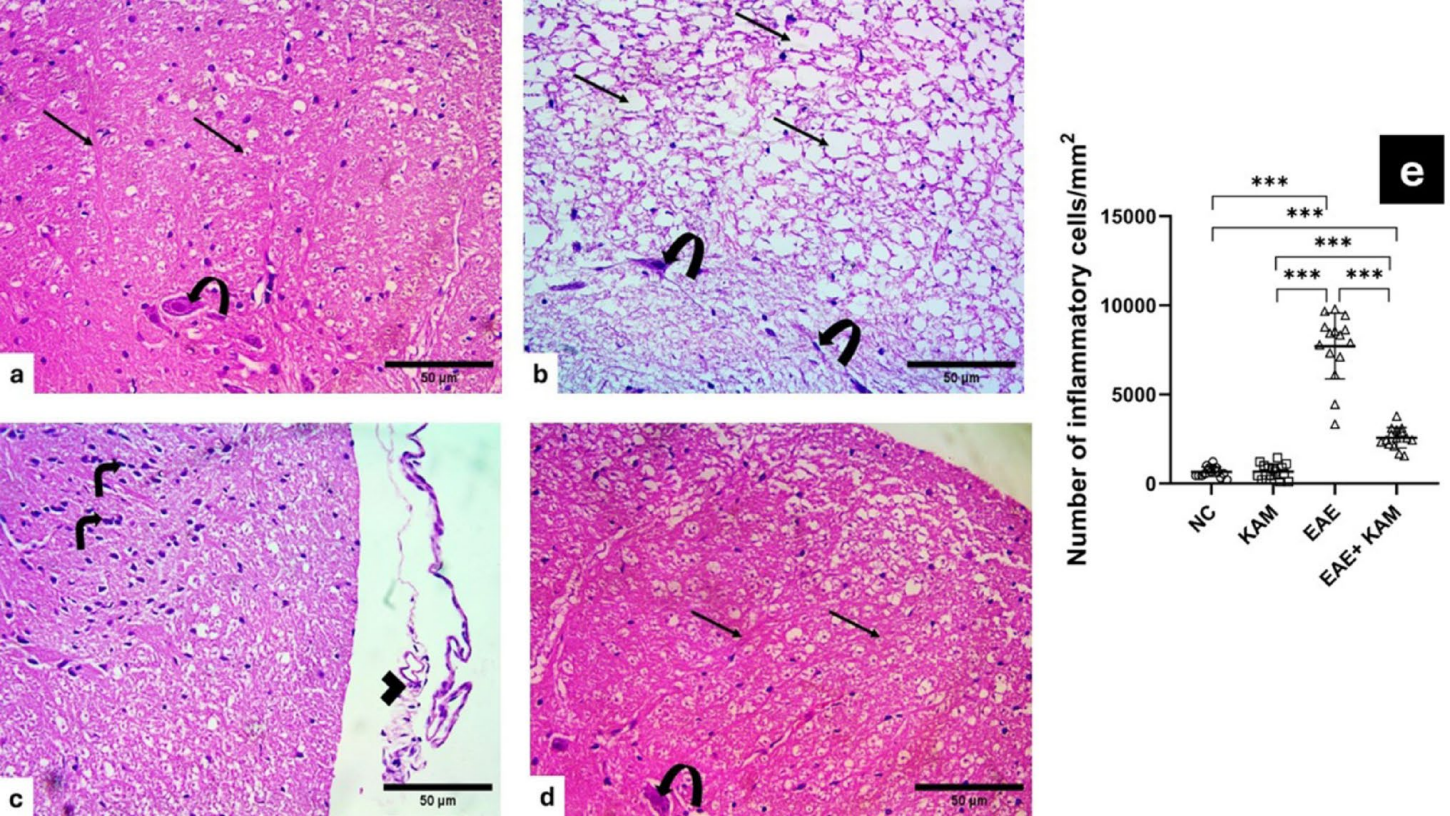
Effect of KAM (50 mg/kg) treatment on the spinal cord histopathological alteration in the EAE model of MS. **a** Control of the spinal cord shows neuronal cell bodies within the grey matter (curved arrow) and myelinated axons within the white matter (arrows). **b** EAE group with irregular neuronal cell bodies with dark dense nuclei (curved arrows) within the grey matter and white matter with marked loss of myelinated axons (arrows). **c** EAE group shows focal inflammatory cellular infiltration within the white matter (angled arrows) and around blood capillaries within the separated meninges (arrowhead). **d** The KAM-treated group shows restoration of the myelinated axons within the white matter (arrows) and normal neuronal cell bodies within the grey matter. **e** Comparison between the studied groups as regards the mean number of inflammatory cells/mm² in spinal cord sections. (Magnification: X400, H&E, scale bar: 50 µ). NC: normal control; KAM: kaempferol; EAE: experimental autoimmune encephalomyelitis

Examination of spinal cord sections from control groups revealed neuronal cell bodies with prominent nuclei within the grey matter and myelinated axons within the white matter (Fig. 3a). EAE group showed neuronal cell bodies with dark dense nuclei within the grey matter, a marked loss of myelinated axons within the white matter in some sections (Fig. 3b) and focal others with inflammatory cellular infiltration within the white matter and around blood capillaries in separated meninges (Fig. 3c) and depicted an extremely significant increase in the mean number of inflammatory infiltrates/mm^2^ as compared to the control group (F (3,56) = 167, *P* < 0.0001, Fig. 3e). KAM treated group depicted marked improvement and restoration of myelinated axons within the white matter and a significant decrease in the number of inflammatory infiltrates as compared to the EAE group (Fig. 3d&e).

### KAM Mitigated TEM Abnormalities in the Brain and Spinal Cord of EAE Mice

**Fig. 4:**
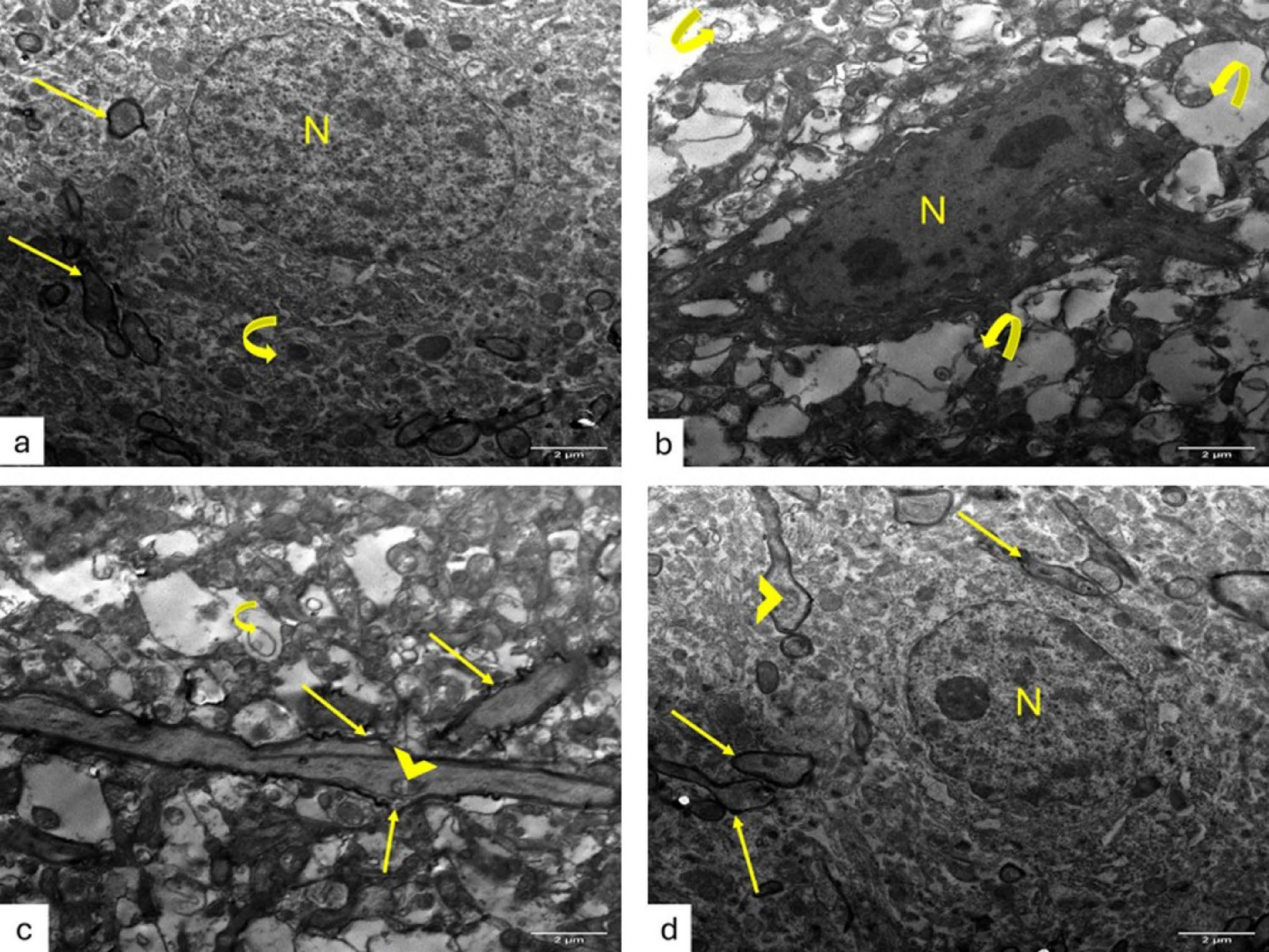
Effect of KAM (50 mg/kg) treatment on brain (cerebral cortex) TEM changes in the EAE model of MS. **a** control cerebral cortex showing pyramidal neuron with euchromatic nucleus (N) and surrounded by neuropil composed of both unmyelinated (curved arrow) and myelinated axons (arrows). **b** EAE group with marked deterioration, as most of the neurons appear irregular with irregular nuclei (N), surrounded by disturbed unmyelinated axons containing mitochondria with destroyed cristae (curved arrows). **c** EAE group shows myelinated axons with disturbed, split thin myelin (arrows) and mitochondria with destroyed cristae within the myelinated (arrowhead) and the unmyelinated (curved arrow) axons. **d** The KAM-treated group shows neuron with euchromatic nucleus (N) and is surrounded by intact neuropil of myelinated axons (arrows) with few with mitochondria with cristolysis (arrowhead). (Magnification: X2000, scale bar: 2 µ, TEM). NC: normal control; KAM: kaempferol; EAE: experimental autoimmune encephalomyelitis

TEM examination of ultrathin sections from the cerebral cortex of control groups revealed pyramidal neurons with euchromatic nuclei and surrounded by an intact neuropil composed of unmyelinated and myelinated axons (Fig. 4a). EAE group examination showed severely disturbed architecture with abnormal neurons with irregular nuclei surrounded by marked vacuolation of the neuropil, abnormal unmyelinated axons contained mitochondria with destroyed cristae. Myelinated axons within the neuropil appeared with irregularly split myelin and contained mitochondria with destroyed cristae (Fig. 4b&c). Interestingly, neurons within the KAM-treated group appeared with euchromatic nuclei, surrounded by an intact neuropil of myelinated axons and few appeared containing mitochondria with cristolysis (Fig. 4d).

**Fig. 5:**
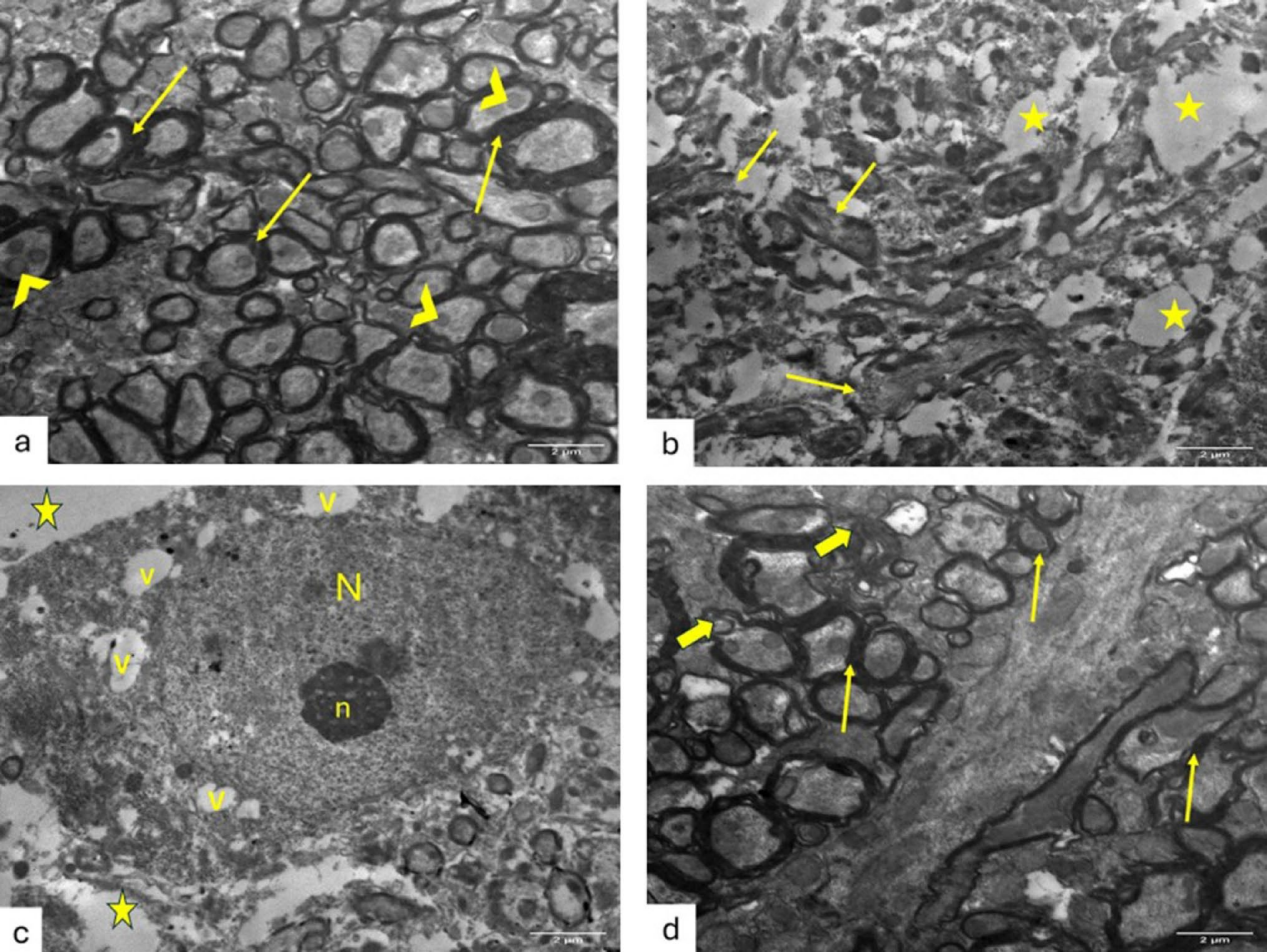
Effect of KAM (50 mg/kg) treatment on the spinal cord TEM changes in the EAE model of MS. **a** The control group showing white matter of the spinal cord with crowded myelinated axons (arrows) containing normal mitochondria (arrowheads). (**b** & **c**) EAE group with severe deterioration of myelinated axons, which appear with either thin discontinuous myelin (arrows) or even loss of myelinated axons (stars). Neurons appear with an euchromatic nucleus (N), a prominent nucleolus (n), vacuolated cytoplasm (v), and are surrounded by vacuolated neuropil (stars). **d** The KAM-treated group shows improvement in the presence of myelinated axons (arrows), except some of them are still surrounded by split myelin (thick arrows). (Magnification: X2000, scale bar: 2 µ, TEM). NC: normal control; KAM: kaempferol; EAE: experimental autoimmune encephalomyelitis

Ultrathin sections examination from the spinal cord of control groups depicted crowded myelinated axons within the white matter containing normal mitochondria (Fig. 5a). The EAE group showed marked disturbance of the architecture with loss of myelinated axons or the presence of thin, split myelin or even discontinuous myelin and mitochondria with destroyed cristae (Fig. 5b&c). KAM administration improves myelination and the presence of myelinated axons (Fig. 5d).

### KAM Effectively Attenuated Ferroptosis and Oxidative Damage in the Brain and Spinal Cord of EAE Mice

**Fig. 6:**
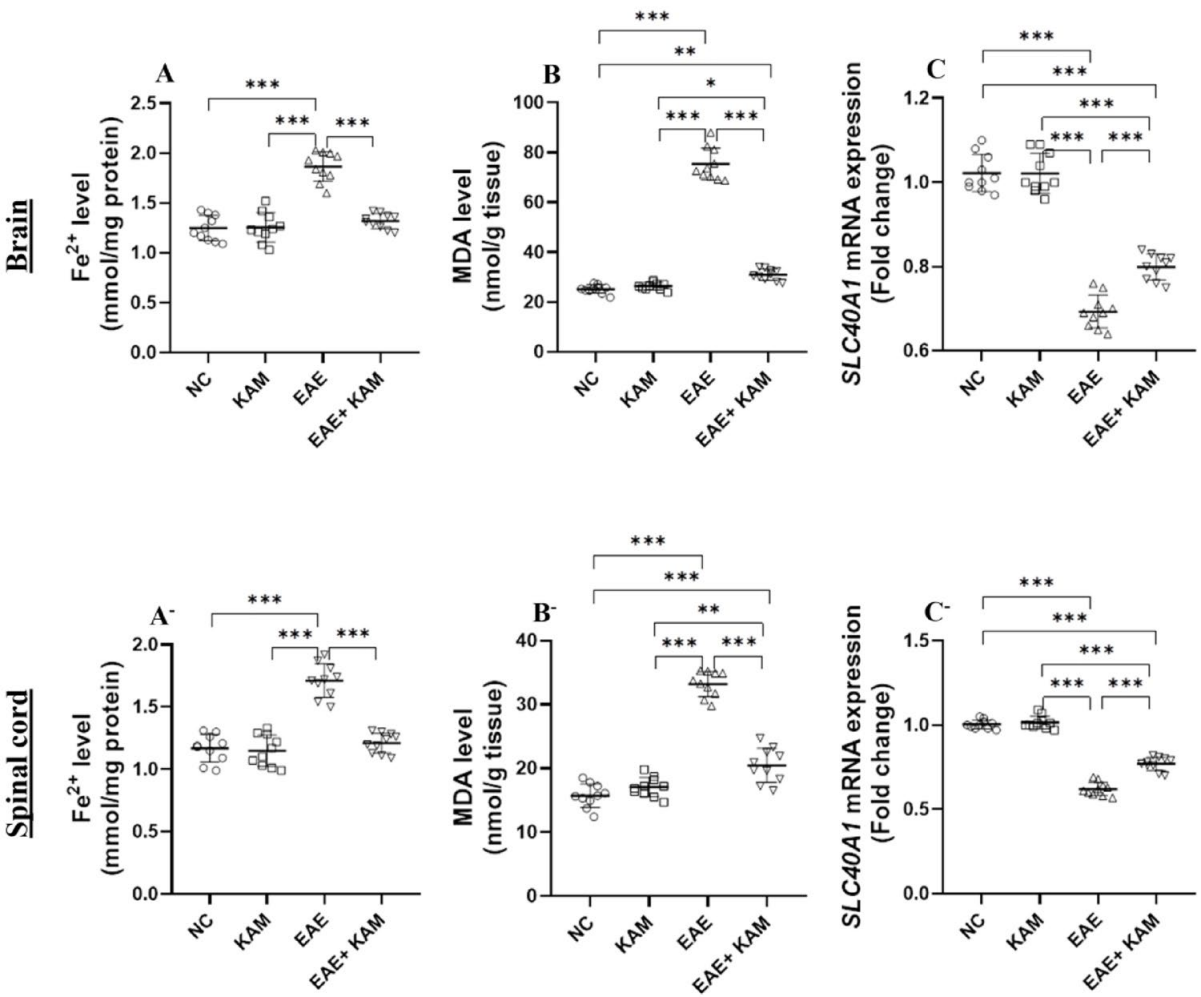
Effect of KAM (50 mg/kg) treatment on the brain and spinal cord Fe^+ 2^ level (**A** and **A**^−^, respectively); MDA level **B** and **B**^−^, respectively); and *SLC40A1* (ferroportin1-encoding gene) mRNA expression (**C** and **C**^−^, respectively) in an EAE model of MS. Values were presented as mean ± SD (*n* = 10). *p < 0.05; **p < 0.01; and ***p < 0.001, by one-way ANOVA followed by Tukey’s post hoc test. Fe^+ 2^: ferrous iron; MDA: malondialdehyde; SLC40A1: solute carrier family 40 member 1; NC: normal control; KAM: Kaempferol; EAE: experimental autoimmune encephalomyelitis

Recent studies have revealed that ferroptosis, a nonapoptotic type of regulated cell death initiated by iron accumulation and lipid peroxidation-mediated stress, accelerates oligodendrocyte loss, demyelination, and neurodegeneration, significantly contributing to MS pathogenesis [7]. To explore whether the neuroprotective impact of KAM was related to the suppression of ferroptosis, we evaluated key ferroptosis markers, including Fe²⁺, MDA, Fpn1, SLC7A11, GSH, and GPX4. Our findings revealed a drastic elevation in the level of intracellular Fe^2+^ in the brain and spinal cord of MOG^35–55^-immunized EAE mice, which was effectively reduced by KAM treatment (F (3, 36) = 54.4, *P* < 0.0001, Fig. 6A and F (3, 36) = 54.6, *P* < 0.0001, Fig. 6A^−^, respectively). Similarly, the brain and cord tissue levels of MDA (F (3, 36) = 442, *P* < 0.0001, Fig. 6B and F (3, 36) = 442, *P* < 0.0001, Fig. 6B^−^, respectively) were greatly elevated in EAE mice compared to the NC group, but these levels were obviously mitigated by KAM (50 mg/kg) administration in the EAE + KAM group. While the EAE group displayed a notable decline in the mRNA levels of the Fpn1-encoding gene (*SLC40A1*) in the brain and spinal cord tissues from untreated MOG^35–55^-immunized mice, meanwhile KAM (50 mg/kg) treatment considerably enhanced *SLC40A1* relative expression in the CNS from the EAE + KAM group compared to the EAE-untreated group (F (3, 36) = 162, *P* < 0.0001, Fig. 6C and F (3, 36) = 273, *P* < 0.0001, Fig. 6C^−^). These findings suggest that KAM may inhibit iron overload by promoting Fpn1-mediated iron efflux.

**Fig. 7:**
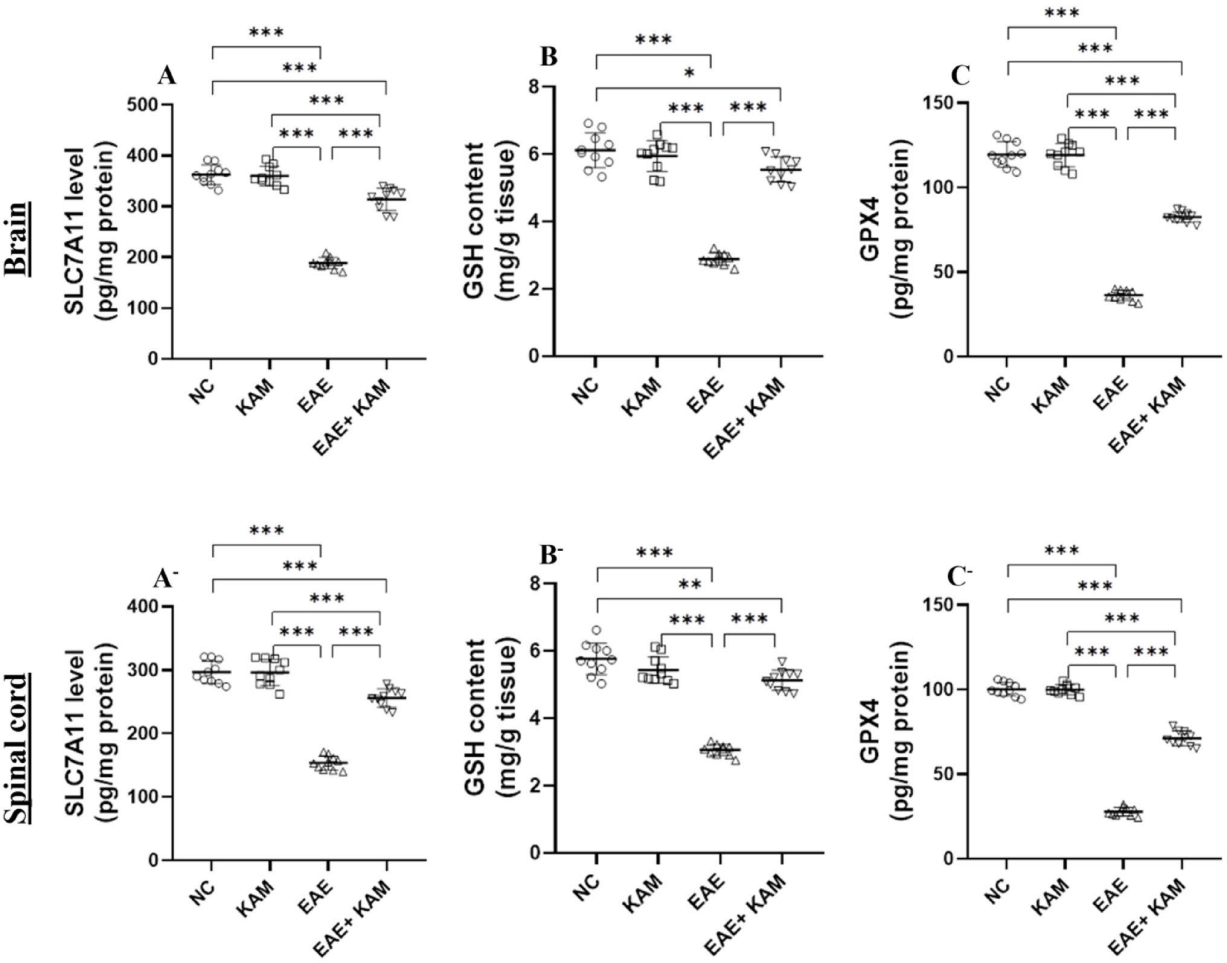
Effect of KAM (50 mg/kg) treatment on the brain and spinal cord SLC7A11 level (**A** and **A**^−^, respectively); GSH level (**B** and **B**^−^, respectively); and GPX4 level (**C** and **C**^−^, respectively) in an EAE model of MS. Values were presented as mean ± SD (*n* = 10). *p < 0.05; **p < 0.01; and ***p < 0.001, by one-way ANOVA followed by Tukey’s post hoc test. SLC7A1: solute carrier family 7 member A1; GSH: reduced glutathione; GPX4: glutathione peroxidase 4; NC: normal control; KAM: Kaempferol; EAE: experimental autoimmune encephalomyelitis

The levels of SLC7A11 in the brain and spinal cord of EAE mice were remarkably lower compared to those of the NC and KAM groups, whereas their levels were substantially increased in the EAE group treated with KAM (50 mg/kg) compared to those of the EAE group (F = 199, *P* < 0.0001, Fig. 7A and F = 172, *P* < 0.0001, Fig. 7A^−^). Moreover, the content of antioxidant marker GSH (F = 142, *P* < 0.0001, Fig. 7B and F = 121, *P* < 0.0001, Fig. 7B^−^) and the level of GPX4 (F = 515, *P* < 0.0001, Fig. 7C and F = 960, *P* < 0.0001, Fig. 7C^−^) were obviously reduced in the brain and spinal cord of MOG^35–55^-immunized EAE mice, but these reductions were effectively reversed by KAM treatment in the EAE + KAM group versus the EAE-only group. Taken together, these results indicate that KAM inhibits neuronal ferroptosis in the EAE mouse model of MS by restoring the SLC7A11/GSH/GPX4 axis.

### **KAM Significantly Suppressed Neuroinflammation in the Brain and Spinal Cord of EAE Mice**

**Fig. 8:**
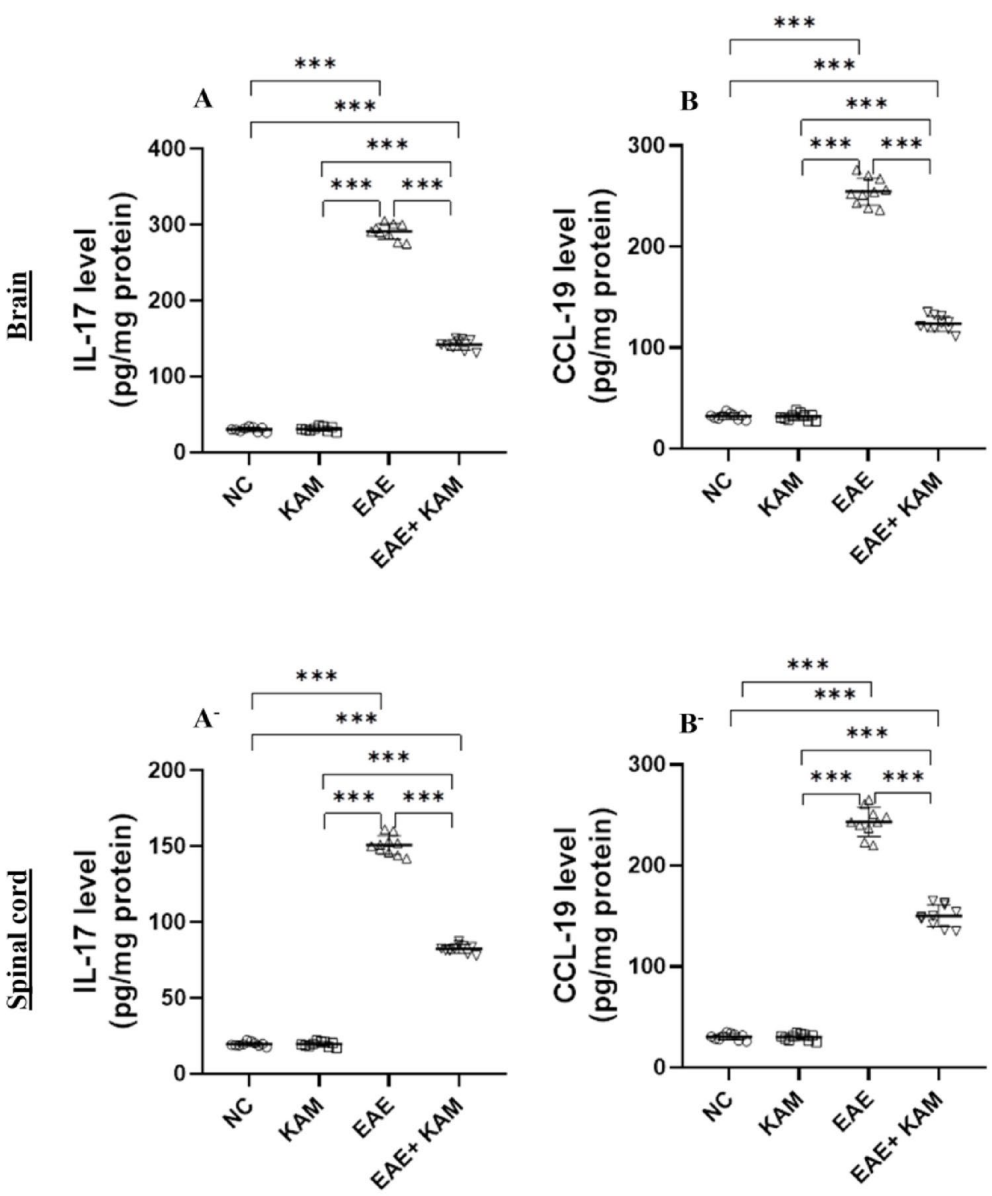
Effect of KAM (50 mg/kg) treatment on the brain and spinal cord IL-17 (**A** and **A**^−^, respectively) and CCL-19 (**B** and **B**^−^, respectively) levels in an EAE model of MS. Values were presented as mean ± SD (*n* = 10). ***p < 0.001, by one-way ANOVA followed by Tukey’s post hoc test. IL-17: interleukin-17; CCL-19: C-C motif chemokine ligand-19; NC: normal control; KAM: Kaempferol; EAE: experimental autoimmune encephalomyelitis

Dysregulation of pro-inflammatory mediators (cytokines and chemokines) contributes significantly to the development of immune-mediated neuroinflammation, demyelination, and neurodegeneration in the CNS, as well as in the context of MS [25]. Therefore, we assessed the levels of IL-17 and CCL-19 in the brains and spinal cords of all tested groups using the ELISA technique. It was found that the brain and spinal cord levels of proinflammatory cytokine IL-17 (F = 3154, *P* < 0.0001, Fig. 8A and F = 3031, *P* < 0.0001, Fig. 8A^−^, respectively) and chemokine CCL-19 (F = 1720, *P* < 0.0001, Fig. 8B and F = 1249, *P* < 0.0001, Fig. 8B^−^, respectively) in EAE mice were remarkably increased compared to those in the NC and KAM groups. It was noteworthy that their levels were considerably reduced in the EAE + KAM group compared to those in the EAE group. This highlighted the immunomodulatory and anti-inflammatory potential of KAM, which may contribute to its neuroprotection.

### KAM Notably Upregulated CNTF Through Activation of the cAMP/CREB Signaling Pathway in the Brain and Spinal Cord of EAE Mice

**Fig. 9:**
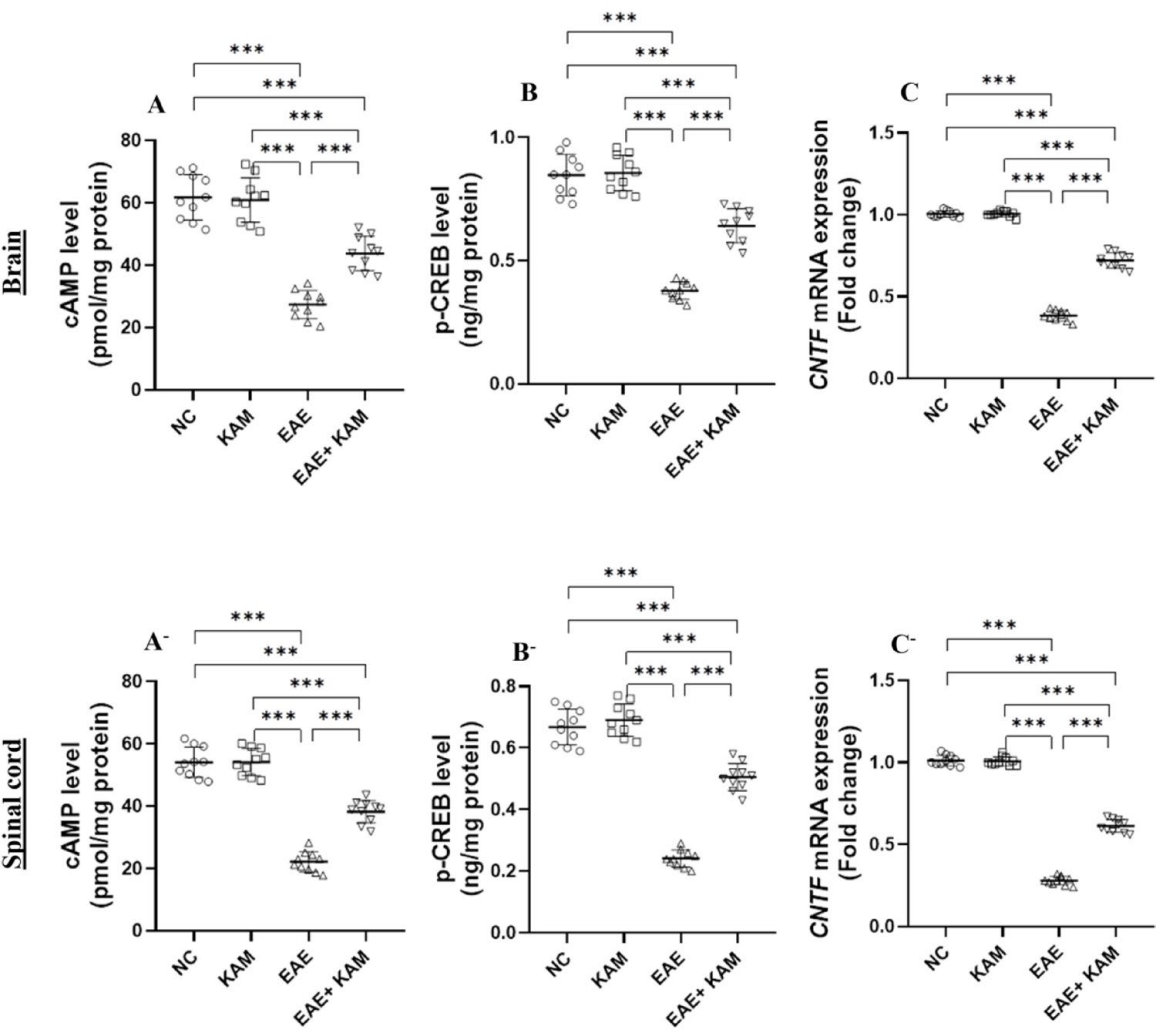
Effect of KAM (50 mg/kg) treatment on the brain and spinal cord cAMP level (**A** and **A**^−^, respectively); p-CREB level (**B** and **B**^−^, respectively); and *CNTF* mRNA expression (**C** and **C**^−^, respectively) in an EAE model of MS. Values were presented as mean ± SD (*n* = 10). ***p < 0.001, by one-way ANOVA followed by Tukey’s post hoc test. p-CREB: phosphorylated-cAMP response element binding protein; CNTF: ciliary neurotrophic factor. NC: normal control; KAM: Kaempferol; EAE: experimental autoimmune encephalomyelitis

cAMP activation is pivotal in promoting neuroplasticity, neurotransmission, neuronal survival, and myelination, while suppressing neuroinflammation and immune activation [13]. Indeed, cAMP activation regulates various biomolecules such as CNTF, a neurotrophic cytokine identified as a survival factor for neurons that exhibits effective promyelinating properties [14]. Thus, we clarified whether the cAMP/CREB/CNTF axis may be a potential underlying molecular mechanism of KAM’s neuroprotective activity against EAE. Protein levels of cAMP and its downstream transcription factor, p-CREB, were unequivocally reduced by EAE induction. Controversially, the i.p. injection of KAM (50 mg/kg) counteracted this suppressive effect, as manifested by a noticeable elevation of the brain and cords cAMP (F = 69, *P* < 0.0001, Fig. 9A and F = 142, *P* < 0.0001, Fig. 9A^−^, respectively) and p-CREB (F = 109, *P* < 0.0001, Fig. 9B and F = 195, *P* < 0.0001, Fig. 9B^−^, respectively) levels in the EAE + KAM group compared to the MOG^35–55^-immunized group. These effects coincided with alterations in *CNTF* relative expression, which was substantially downregulated in the brains and cords of EAE mice versus control groups. Compared with the EAE group, KAM treatment (50 mg/kg) considerably increased *CNTF* mRNA levels in the CNS tissue from KAM-treated EAE mice (F = 922, *P* < 0.0001, Fig. 9C and F = 1253, *P* < 0.0001, Fig. 9C^−^, respectively). Collectively, these results demonstrate that KAM promotes the activation of the cAMP/CREB axis and upregulates *CNTF.*

### KAM Effectively Enhanced *miR-367–3p* Expression in the Brain and Spinal Cord of EAE Mice

**Fig. 10:**
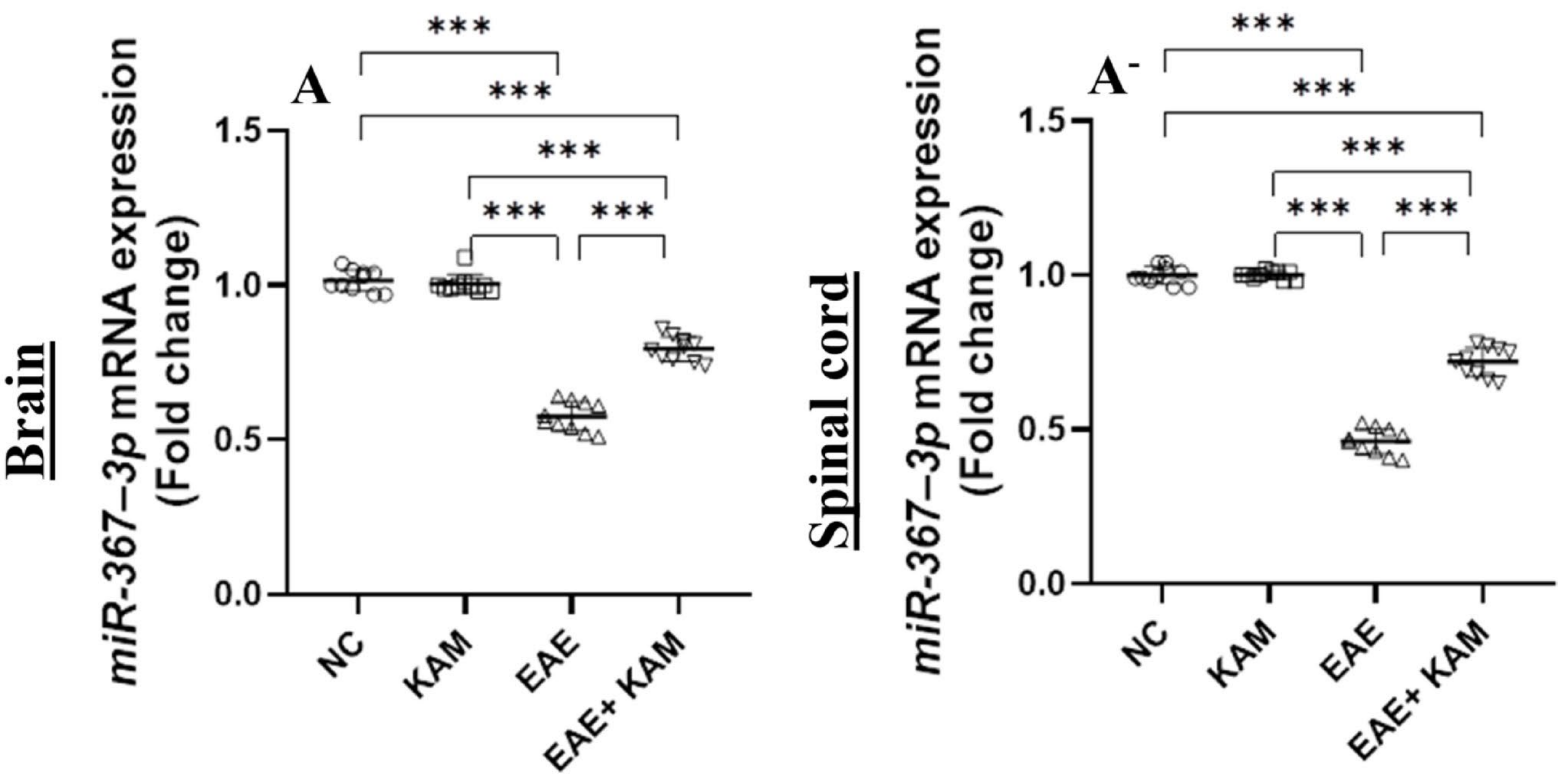
Effect of KAM (50 mg/kg) treatment on the brain and spinal cord *miR-367–3p* mRNA expression (**A** and **A**^−^, respectively) in an EAE model of MS. Values were presented as mean ± SD (*n* = 10). ***p < 0.001, by one-way ANOVA followed by Tukey’s post hoc test. NC: normal control; KAM: Kaempferol; EAE: experimental autoimmune encephalomyelitis

According to recently published studies, *miR-367–3p* upregulation negatively regulates microglia activation, inflammatory response, and ferroptosis in various CNS disorders such as ischemic stroke [16] and MS [18]. In this regard, upregulation of *miR-367-3p* may constitute a promising therapeutic candidate for MS. Consequently, we planned to examine its gene expression in all studied mouse groups. Brain and spinal cord *miR-367–3p* gene expression levels were remarkably downregulated in the MOG^35–55^-immunized EAE group compared to the NC group (F = 289, *P* < 0.0001, Fig. 10A and F = 542, *P* < 0.0001, Fig. 10A^−^, respectively). In contrast, its mRNA levels were effectively upregulated upon KAM treatment (50 mg/kg) in the KAM-treated EAE mice compared to EAE-untreated mice. These findings provide a novel molecular mechanism for KAM’s neuroprotective potential.

### KAM Promoted Myelination in the Brain and Spinal Cord of EAE Mice

**Fig. 11:**
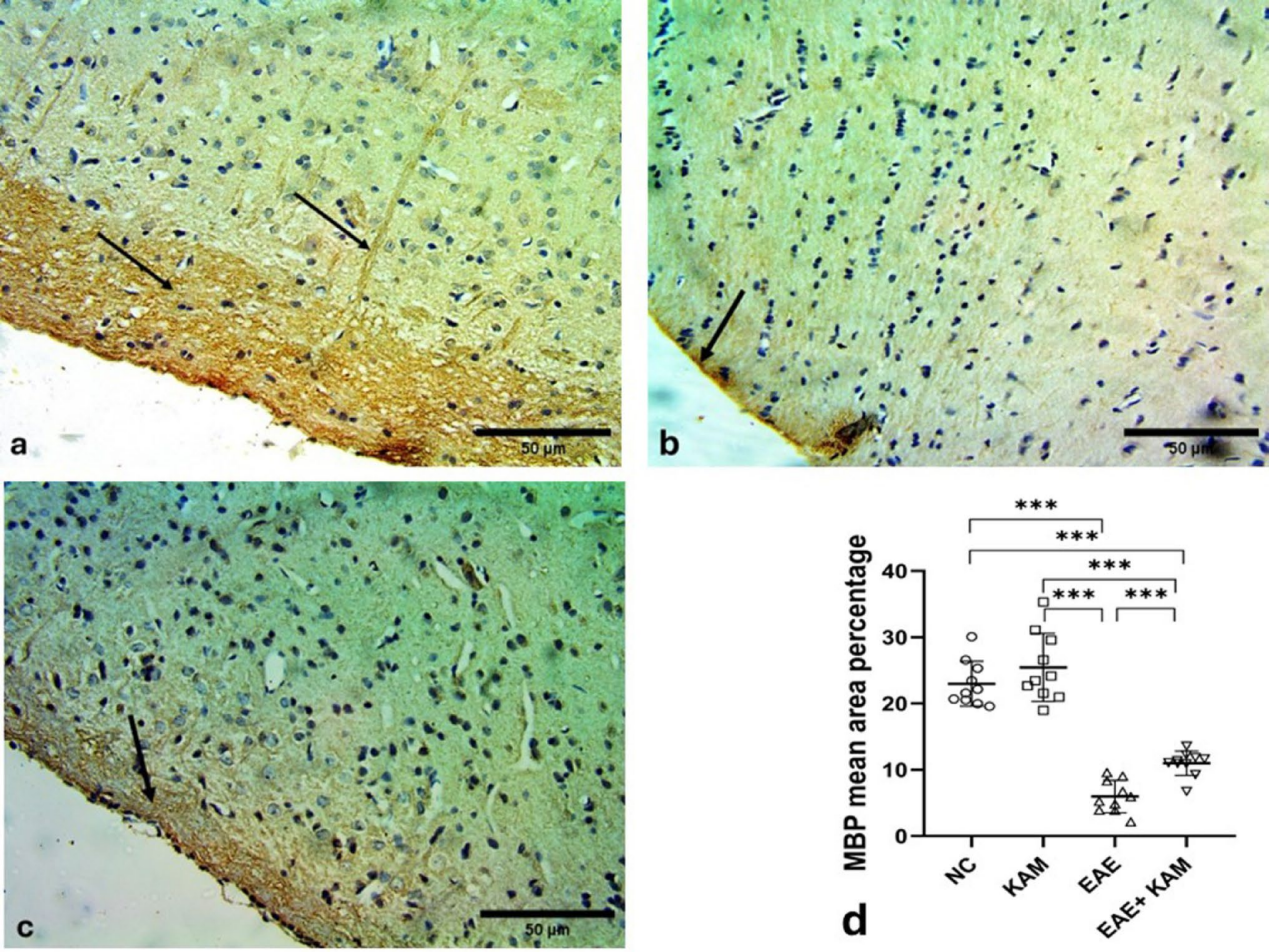
Effect of KAM (50 mg/kg) treatment on brain (cerebral cortex) MBP immunohistochemistry in the EAE model of MS. **a** control cerebral cortex showing strong widespread positive immunoreaction of MBP in myelinated axons within the molecular layer (arrows). **b** EAE group with limited weak positive immunoreaction of MBP in myelinated axons within the molecular layer (arrow). **c** The KAM-treated group had a moderate positive immunoreaction of MBP in myelinated axons within the molecular layer (arrow). **e** comparison between the studied groups as regards the mean area percentage of MBP in cerebral cortex sections. (Magnification: X400, scale bar: 50 µ). MBP: myelin basic protein; NC: normal control; KAM: kaempferol; EAE: experimental autoimmune encephalomyelitis

Examination of cerebral cortex sections stained immunohistochemically with MBP antibody showed strong positive immunoreaction within the molecular layer (Fig. 11a). While the EAE-induced group showed a weak reaction in the remaining myelinated axons within the molecular layer (Fig. 11b) and an extremely significant decrease in the mean area percentage of MBP as compared to the control group (F (3,36) = 74.4, *P* < 0.0001, Fig. 11d). Noteworthy, the KAM-treated group depicted a moderate positive reaction for MBP and a significant increase in the mean area percentage of MBP as compared to the EAE group, which indicated it enhanced myelination (Fig. 11c&d).

**Fig. 12:**
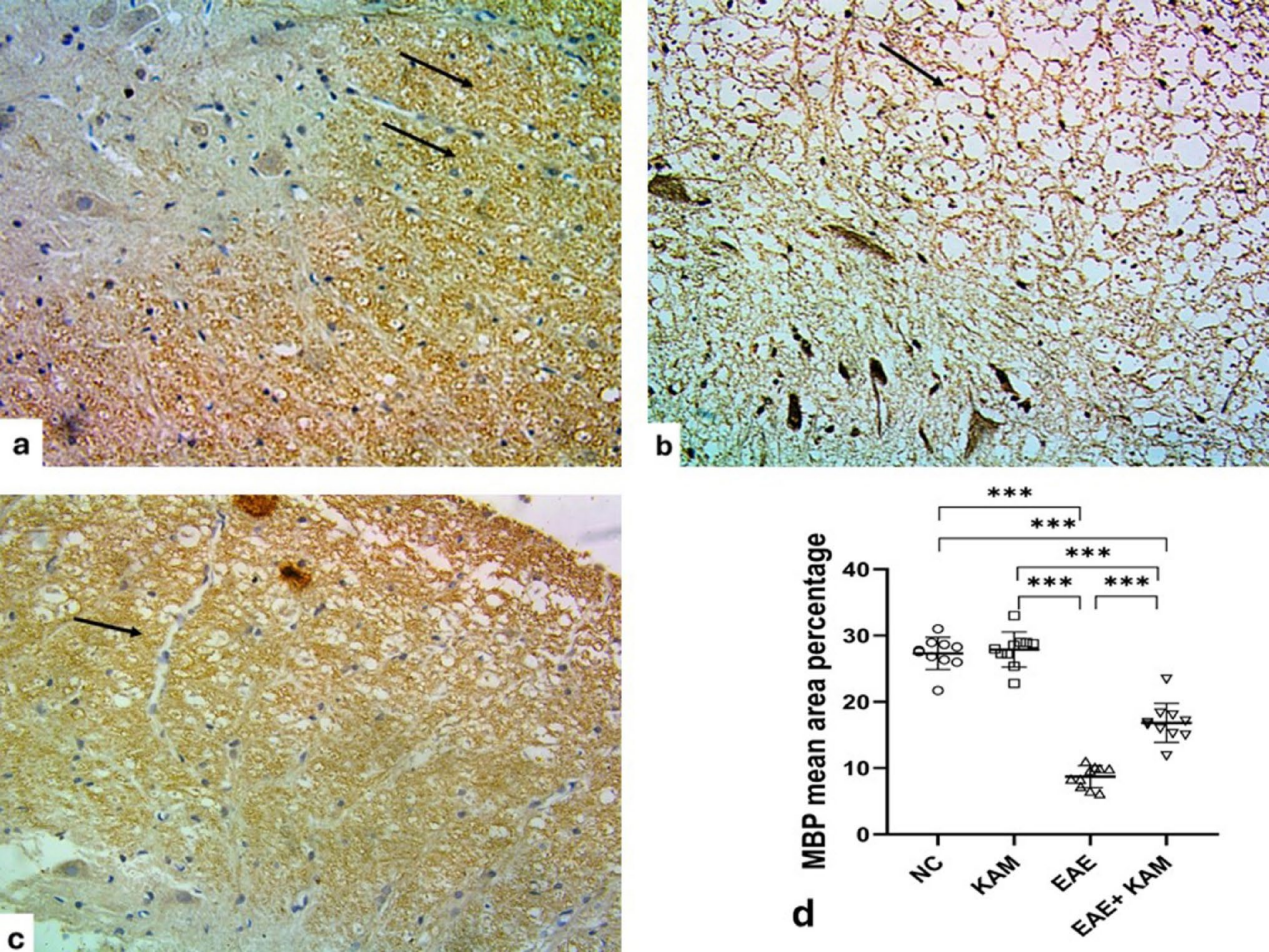
Effect of KAM (50 mg/kg) treatment on the spinal cord MBP immunohistochemistry in the EAE model of MS. **a** The control spinal cord shows strong widespread positive immunoreaction of MBP in myelinated axons within the white matter (arrows). **b** EAE group with limited weak positive immunoreaction of MBP in the remnants of myelinated axons within the white matter (arrow). **c** The KAM-treated group had a moderate positive immunoreaction of MBP in myelinated axons within the white matter (arrow). **d** Comparison between the studied groups as regards the mean area percentage of MBP in spinal cord sections. (Magnification: X400, scale bar: 50 µ). MBP: myelin basic protein; NC: normal control; KAM: kaempferol; EAE: experimental autoimmune encephalomyelitis

Sections from the spinal cord showed strong positive immunoreaction to MBP within the white matter (Fig. 12a). The EAE group showed weak immunoreaction in the remaining myelin within the white matter (Fig. 12b) and an extremely significant decrease in the mean area percentage of MBP as compared to the control group (F (3,36) = 138, *P* < 0.0001, Fig. 12d). KAM treatment depicted a moderate immunoreaction to MBP and a significant increase in the mean area percentage of MBP as compared to the EAE group as an indicator of its effect on the improvement of myelination (Fig. 12c&d).

## Discussion

The current study highlighted the neuroprotective potential of KAM in the MOG^35–55^ peptide-induced EAE mouse model of MS. This neuroprotection appears to be mediated through targeting neuronal ferroptosis and modulating the cAMP/CREB/CNTF signaling axis, potentially via regulation of *miRNA-367-3p* expression. Our findings demonstrated that KAM effectively reduces EAE progression by alleviating neuronal ferroptosis, neuroinflammation, and axonal demyelination, while restoring redox balance and promoting remyelination. These effects are supported by both histopathological observations and biochemical analyses. These results are consistent with previous studies reporting the anti-inflammatory, antioxidant, and immunomodulatory properties of KAM in neurological disorders [21–23].

Research consistently indicates a significant gender disparity in EAE susceptibility. Immune responses differ substantially between males and females. The vast majority of studies in animal models have been conducted exclusively on one sex, under the assumption that results would generalize to the other. However, data show a clear female bias in EAE, with reports indicating that approximately 85% of EAE studies use female rodents exclusively. Females not only develop the disease at a rate exceeding 80%, compared to less than 30% in males, but also exhibit earlier disease onset and more severe clinical symptoms [5]. This disparity arises from multifactorial causes, including genetic differences on female sex chromosomes and a hormonal milieu characterized by moderate estrogen and low testosterone levels. This environment permits robust T-cell activation and promotes a strong pro-inflammatory Th1/Th17 bias at disease initiation, which is further amplified by factors such as prolactin and pro-inflammatory microbiota. In contrast, males are relatively protected by higher androgen levels, which foster central nervous system tolerance by suppressing inflammatory T-cell responses, combined with a generally less reactive innate and adaptive immune baseline and increased expression of the autoimmune regulator (Aire) in the thymus, providing additional protection against autoimmune diseases [33].

Emerging evidence underscores ferroptosis as a pivotal mechanism in MS pathogenesis and progression, particularly contributing to the degeneration of oligodendrocytes, the myelinating cells [7]. Recent studies have highlighted iron dysregulation as a central feature of ferroptosis, characterized by excessive deposition of redox-active iron and activation of lipid peroxidation cascades. These processes lead to fragmentation of cell membranes and ultimately cause cell death, impairing myelination [34]. Consistent with previous research [35], the findings of this study demonstrate that ferroptosis is evident in the EAE model, as indicated by Fe²⁺ overload, GSH depletion, MDA augmentation, and SLC7A11 and GPX4 diminution.

Remarkably, in the context of MS, ferroptotic neurons release mediators that activate T-cell receptor pathways, leading to elevated mRNA levels of IL-2 and interferon-γ in T cells. These activated T cells exacerbate immune cell–mediated axonal demyelination [36, 37]. Moreover, CD4^+^ T cells themselves undergo ferroptosis, which enhances their activation and promotes differentiation into Th17 cells, resulting in increased IL-17 secretion. IL-17 subsequently disrupts the blood-brain barrier, recruits monocytes, and activates microglia, which release pro-inflammatory cytokines, augmenting production of neurotoxins and other inflammatory molecules [38–40].

Additionally, activated microglia and infiltrating macrophages in MS lesions produce CCL-19, a chemokine that attracts CCR7^+^ T cells and dendritic cells to inflammatory sites, orchestrating MS pathogenesis [41]. This establishes a feedback loop of sustained immune activation and chronic central nervous system inflammation, ultimately leading to oligodendrocyte death and intensified demyelination [14]. Furthermore, iron-driven lipid peroxidation destabilizes the myelin sheath, rich in polyunsaturated fatty acids (PUFAs), accelerating axonal degeneration [42]. Supporting this notion, a marked increase in both IL-17 and CCL-19 levels was observed in the untreated EAE group compared to the treated groups.

In the current report, the downregulation of the Fpn1-encoding gene (*SLC40A1*) observed in EAE mice may represent a critical factor contributing to the iron overload detected. This downregulation disrupts Fpn1 trafficking to the plasma membrane, impairing cellular iron efflux and amplifying the labile iron pool (LIP), thereby exacerbating iron toxicity under inflammatory conditions [11, 12]. Biochemically, an excessive accumulation of Fe^2+^ within the cell activates Fenton’s reaction, generating reactive oxygen species like hydroxyl radicals (OH^•^). These radicals subsequently abstract hydrogen from polyunsaturated lipids, creating lipid radicals (L•) that propagate the process of lipid peroxidation (shown by MDA elevation and GSH depletion in the EAE group), evoking oligodendrocyte ferroptosis and subsequent axonal demyelination [43, 44]. This research highlighted the involvement of iron exporter protein Fpn1 in the molecular mechanisms underlying MS pathogenesis, marking the first evidence of its role in the disease’s development.

Noticeably, KAM, a natural bioflavonoid with well-established anti-inflammatory, free radical–scavenging, and antioxidant properties, demonstrated significant neuroprotective effects in the present study by counteracting ferroptosis. This was evidenced by a marked reduction in Fe²⁺ overload and the lipid peroxidation product, MDA, in the EAE + KAM group. Furthermore, KAM treatment enhanced neuronal resistance to ferroptotic cell death by upregulating key anti-ferroptosis proteins, including SLC7A11, GSH, and GPX4. These findings are consistent with previous reports by Yuan et al. and Li et al. [45, 46], who demonstrated KAM’s anti-ferroptotic activity through mechanisms involving iron chelation, GPX4/SLC7A11 elevation, and attenuation of oxidative stress. Such results align with the recognized role of ferroptosis modulation in neuroprotection.

As a novel aspect of this study, it is the first to investigate KAM’s anti-ferroptotic signature in the EAE model of MS. Our results revealed that KAM treatment significantly upregulated *Fpn1* gene expression, which may partly explain its role in reducing intracellular Fe²⁺ levels by promoting iron export and hampering the expansion of the LIP. Concurrently, KAM appears to enhance neuronal tissue resistance to ferroptotic cell death, primarily through the activation of key ferroptosis-regulatory genes, notably *miRNA-367-3p*.

The proposed mechanism suggests that KAM upregulates *miRNA-367-3P*, which targets SLC7A11 by directly binding to the 3’ untranslated region (3’UTR) of enhancer of zeste homolog 2 (EZH2) mRNA. This interaction inhibits EZH2 protein expression through mRNA degradation or translational repression. Since EZH2 serves as the catalytic core of Polycomb Repressive Complex 2 (PRC2), its reduction substantially diminishes PRC2’s enzymatic activity. Consequently, PRC2 deposits fewer methyl groups on histone H3 at lysine 27 (H3K27me3), a repressive mark specifically at the *SLC7A11* promoter. The loss of this repressive mark de-represses (activates) *SLC7A11* transcription, leading to increased SLC7A11 protein expression [18]. The cystine/glutamate antiporter SLC7A11 facilitates cystine uptake, which is rapidly reduced to cysteine, an essential precursor for GSH synthesis. By providing the essential substrate for GPX4 (a major ferroptosis inhibitor), it will be able to detoxify lipid peroxides and protect neural cells from oxidative damage and ferroptosis death, thus alleviating EAE symptoms [44].

Additionally, miRNA-367-3P indirectly enhances nuclear factor erythroid 2-related factor 2 (Nrf2) through suppressing the G protein-coupled receptor, family C, group 5, member A (*GPRC5A*), a novel miR-367-3p candidate target gene [16], resulting in a reciprocal elevation in intracellular cAMP concentration [47]. Subsequently, this increases AMPK activity, which in turn phosphorylates and activates Nrf2. Reinforcing this perspective, He et al. [48] reported that miRNA-367-3P effectively inhibited Kaep1 expression while enhancing the protein levels of Nrf2 and its downstream heme-oxygenase 1 in an ischemia-reperfusion injury cell model of ischemic stroke. Activated Nrf2 then upregulates glutathione-producing genes, *SLC7A11*, *GPX4*, and *SLC40A1* (Fpn1-encoding gene), possibly explaining Fpn1’s upregulating effect of KAM. Consistent with our findings, recent studies highlight that KAM activates Nrf2, thereby upregulating SLC7A11 and GPX4 to mitigate lipid peroxidation and iron overload in neuronal models of ischemic stroke [49]. For the first time, the significant upregulation of the Fpn1-encoding gene and restoration of the SLC7A11/GPX4/GSH signaling pathway via modulating *miRNA-367-3P* expression represent a new mechanistic insight into KAM’s anti-ferroptotic and neuroprotective potential in the MOG^35–55^ peptide-induced EAE model of MS.

Recently published studies have pointed to the effects of cAMP on stimulating neurite outgrowth and preserving neuronal survival [13]. At the same time, increased cAMP levels boosted axonal regeneration by modulating myelin-associated inhibitors [50]. Furthermore, activating the cAMP/CREB signaling pathway promoted oligodendrocyte proliferation and myelination and exerted neuroprotective properties against sevoflurane-induced neurotoxicity [51]. Confirming the results of a previous study by Thaweewattanodom et al. [52], our data indicated that KAM treatment increased cAMP and p-CREB levels in EAE-treated mice, thereby activating the cAMP/CREB transduction axis. cAMP, in turn, stimulates protein kinase A (PKA), which phosphorylates CREB. This activation promotes neurite outgrowth and myelination while inhibiting microglial activation, neuroinflammation, T cell proliferation, and T cell infiltration into the CNS [13, 50]. Accordingly, this provides an additional molecular mechanism underlying KAM’s neuroprotective potency against experimentally induced MS.

**Fig. 13:**
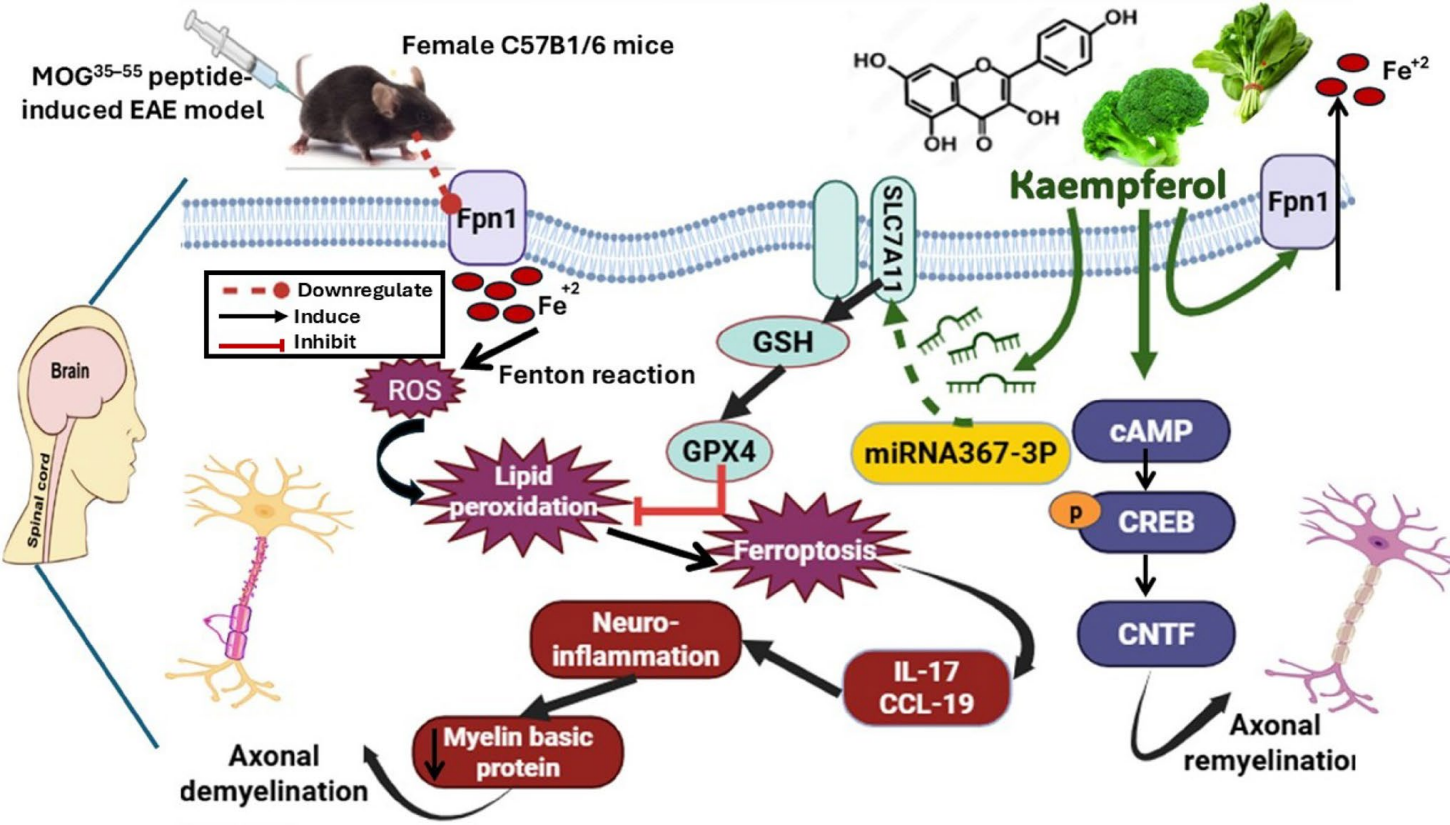
The proposed mechanisms underlie Kaempferol’s neuroprotective effect in the experimental autoimmune encephalomyelitis (EAE) model of multiple sclerosis

Crucially, for the first time, KAM treatment was associated with significant upregulation of *CNTF*, identifying CNTF as a potentially effective target for KAM neuroprotection. CNTF plays an essential role in promoting oligodendrocyte survival and maturation and subsequently enhanced myelination to protect against nerve damage in MS [14]. According to a former study, CNTF treatment induced remyelination, suppressed microgliosis, and reduced T-cell infiltration in EAE [14]. On the same line, unlike the EAE group, the KAM-treated group revealed notable upregulation of *CNTF* expression along with effective restoration of MBP immunoreactivity in brain and spinal cord tissues, documenting KAM’s neurotrophic effect. MBP is a vital biomarker of myelin integrity selectively expressed by mature oligodendrocytes, which plays a crucial role in the myelination process, as it comprises 30% of the total CNS myelin content [14, 51]. Interestingly enough, former research studies have shown that the PKA-CREB pathway enhances CNTF gene expression in astrocytes by activating a CREB-binding site located in the gene’s promoter region [25, 53]. Corroborating these findings, this study provides the first evidence that KAM boosts CNTF gene expression via a mechanism involving the cAMP/CREB/CNTF signaling axis. Figure 13 outlines the underlying mechanisms that might account for the positive effects of KAM in the EAE model of MS.

## Conclusion

This study demonstrates that KAM alleviates clinical neurological deficits, histopathological abnormalities, and ultrastructural damage in an EAE mouse model of MS. Importantly, the current findings highlight the disease-modifying potential of KAM through its anti-ferroptotic effects and its ability to diminish oxidative damage and neuroinflammation. Furthermore, KAM exhibits a potent neurotrophic profile by promoting neuronal repair and axonal remyelination. These beneficial effects are likely mediated by enhanced Fpn1-dependent iron extrusion, restoration of the SLC7A11/GSH/GPX4 axis, activation of the cAMP/CREB/CNTF signaling pathway, and upregulation of *miR-367-3p*, a novel downstream effector associated with KAM treatment. Collectively, these findings advance our understanding of MS pathogenesis and support KAM as a promising multi-target therapeutic candidate for neurodegenerative diseases. Additionally, our results lay the groundwork for further in-depth studies to explore the specific role of miR-367-3p in MS pathophysiology and to develop novel therapeutic avenues.

## Study Limitations

This study has several limitations. First, this study was conducted exclusively on female C57BL/6 mice. Given the well-documented sex differences in MS prevalence, progression, and treatment response, future studies should include male animals to evaluate sex-specific effects of KAM treatment. Second, KAM was used at a stated purity of ≥ 90%, slightly below the typically recommended ≥ 95% purity for biological assays. While ≥ 90% purity is commonly accepted and has demonstrated biological activity in comparable studies, potential effects of residual impurities cannot be fully excluded. Future studies utilizing KAM of ≥ 95% purity are warranted to confirm and extend these findings. Third: while KAM treatment was associated with upregulation of miR-367-3p and neuroprotective effects, direct cause–effect relationships remain unconfirmed. Future studies employing miR-367-3p inhibition or knockdown are needed to clarify whether KAM’s therapeutic effects are dependent on miR-367-3p modulation or whether this represents a parallel observation.

## Data Availability

All data generated or analyzed during this study are included in this article.
